# Mental Dynamics, in Relation to the Science of Medicine

**Published:** 1853-10-01

**Authors:** Stanhope Templeman Speer

**Affiliations:** PROFESSOR OF PHYSIOLOGY IN THE UNIVERSITY OF MONTPELLIER; CHELTENHAM


### Original Communications.

### MENTAL DYNAMICS, IN RELATION TO THE SCIENCE OF MEDICINE.

A COURSE OF LECTURES DELIVERED BY M. LORDAT, PROFESSOR OF PHYSIOLOGY IN THE UNIVERSITY OF MONTPELLIER. ARRANGED AND TRANSLATED BY STANHOPE TEMPLEMAN SPEER, M.D., CHELTENHAM.

### Lecture IV.

Gentlemen,—It would appear that the insenescence of the human intellectual principle affords a most potent argument in favour of the duality of our dynamism, and of the essential distinction between this principle and its coadjutor, the vital force. Already may we surmise, from the sentiment of self-consciousness in the one, and its absence in the other, from the ignorance and innate inability of the former, and the primordial energy of the latter, from the inaction of the intellectual principle during *intra-uterine* existence, and the activity of the vital force, from the moment at which the two agencies were called into being; from all this, I repeat, we may already venture to surmise that the two principles cannot be the different attributes of one and the same cause, but must emanate rather from distinct sources, and be united in one common system. After reflecting upon their respective modes of existence, upon their contemporaneous career, upon the contrast afforded by their mutual progression during the latter period of life, in one word, upon the insenescence of the intellectual principle and the infallible decay of the vital force; I can no longer entertain a doubt of the non-identity of the two principles, and, as a consequence, the duality of the human dynamism stands prominently forth, despite of the hypothesis of Stahl.

On the other hand, however, Stahl has clearly demonstrated, that the vital force is not of a physical order. I consider it needless to corroborate his testimony on this point, since they who persist in asserting that life is the consequence of physical and chemical laws, have undoubtedly but little studied the nature and attributes of the vital force, and ignore entirely the arguments of Stahl.

It may be looked upon, I conceive, therefore, as an incontrovertible fact, that the vital force is *not* of a physical order; nor, indeed, does it constitute the essential element, the essence of psychology. To what natural order then does it belong? In what philosophical category must we place biology, or the science of the vital principle?

Biology in general, and more especially human medical biology, is of a *metaphysical* order. But as the term *metaphysics* has been defamed by materialists, and is even still employed by many in an unfavourable acceptation, it becomes necessary to dwell for a moment upon the primitive legitimate value of the expression, in order to rebut the accusations of the ignorant and prejudiced.

I regret here being obliged to defend the term in question, against certain authorities, in other respects worthy of the highest respect, and who, albeit, nurtured in this university, have employed the word *metaphysics* in an erroneous and unjust sense. More particularly do I complain of a passage in the "Vegetable Physiology" of the late De Candolle, to the following effect. "Barthez," says he, "has made of physiology a species of metaphysics, in which all inquiry into the causation of natural phenomena is dispensed with, and in which, as a substitute for explanation, *words, too often devoid of meaning, are liberally made use of.*" I know not whether this shaft be directed against Barthez himself, or against the science of metaphysics in general. In the former case, I hesitate not to assert, that he who discharged it, could not have read a page of Barthez, whose greatest fault lies in having accumulated and given expression to a superfluity of ideas in a very limited number of terms. In the latter case, he must have forgotten the true signification of the word.

The term *metaphysics* has been employed by Aristotle, although it is possible that the sense in which he understood it, may have been somewhat too general for our present notions. I shall therefore confine myself to the definition of that great restorer of the sciences, Bacon; and should I at times decline to adopt precisely his own terms, I will at least endeavour to remain in strict conformity with the spirit of his meaning.

The sciences, then, considered in relation to their material objects, appear capable of being divided into four distinct classes.

The first includes those which are based upon certain propositions, accepted by religious belief (*Theological sciences*). We will not here inquire as to whether these propositions be truths or merely supposititious assertions. Whether the belief be of divine origin, or whether it be erroneous and unfounded.

The second division comprehends those sciences which are deduced from fictitious facts, accepted to a certain extent through the medium of popular belief, but of which it is impossible clearly to demonstrate either the reality or the fabulousness. We may mention, for example, judicial astrology, divination, the cabalistic art, &c. These may all be included under the title of the *Occult Sciences.*

The third class comprises everything that is founded upon conventional supposition, such, for instance, as the science or doctrine of chance. It appears to me that we might include in this category all *hypothetical* probabilities.

The fourth division is the sum total of those sciences which are founded upon the facts and phenomena presented to our view by the universe at large, and from which our intellectual principle seeks to deduce the origin, effects, and *modus operandi* of causality. Strictly speaking, these sciences may be designated as the *Natural Sciences,* inasmuch as their subject matter is derived directly from Nature herself.

The natural sciences, or in other words, the sciences of nature, are those, the elements of which are real, and the actual subjects of which are founded upon reason; consequently all such as are neither revealed, supposititious, nor conventional. Thus mathematics, ethics, politics, may, in this sense, be termed natural.

It would be out of place here to dwell upon the different significations that have been attached to the words physics and physiology. The philological history of their acceptation would be but the history of opinion. Doubtless it might be interesting, but it would be discursive.

In the first place, then, we are not to confound under the title of science every species of information we may chance to possess. Simple facts devoid of reason or sequence, are mere notions, but they do not constitute science; this term must be exclusively reserved for that totality which is formed by the deduction, the causality, and the connexion of actual facts.

*Physics,* properly speaking, comprise a knowledge of the influence exerted by one body upon another, in virtue of its peculiar properties, without the nature of its substantial element being in any degree altered. Thus this same science determines and predicts the consequences which should result from the contact or union of two bodies, the respective properties of which are known, such for instance as their attraction, ponderability, consistence, elasticity, sonoreity, &c.

Among the properties of matter, a distinction has been drawn between *affinity* and attraction, in that the reciprocal tendency is not so much manifested in the mass as in the molecule, and that the resulting contact is not limited to a mere appliance of the one particle to the other, but becomes an actual combination. The phenomena arising from the above cause, constitute the subject matter of *chemistry.*

Those agents now denominated *Imponderable,* such as light, electricity, heat, and magnetism, have never been confounded with the ordinary properties of matter, with resistance, weight, impenetrability, mobility, and such like attributes pertaining to the domain of mechanics; they have as a consequence necessitated separate consideration. Their elicitation and utilization, moreover, having required much ingenuity and research, and numerous extraordinary effects having been obtained through their instrumentality, it has followed, as a result of the same, that the knowledge of these agents has formed an integral portion of what is called *natural or white magic.* About two hundred years ago, it went by the name of Corpuscular Physics. To-day, each imponderable is the subject of special investigation; they together form a section of the science of physics generally.

These sciences, then, constitute the *physical order,* in the catalogue of human acquirements. The characteristics of this order are: that matter and its properties are identical,—that conditions being determined, the results are infallible,—that the properties of a body being always a constant quantity, the effects will be invariably proportional to such properties, and will continue to be so, however often the phenomena be repeated.

Thus, in what is of a *physical order,* matter acts by its whole mass, without reservation, and as a natural consequence of its own peculiarities, without reference to the past or to the future.

Those bodies, denominated living bodies, or in which take place the phenomena of life (and I have already explained the meaning which I attach to that word), act in a totally different manner, and must be classed in a separate category. It is this category which constitutes the *metaphysical order*, and of which Bacon has traced the distinctive characteristics. Listen to him for a moment, but let me first remark that he passes over what had been previously designated as general metaphysics, and at the commencement of the last century, *Ontology*.

Special metaphysics, or metaphysics properly speaking, "constitute a portion of natural philosophy.

"We may say, without deviating from the spirit of antiquity, that physics treat exclusively of that which is included in matter . . . . . . metaphysics regarding things of a more abstract nature. . . . . . We may say, in addition, that the science of physics presupposes in nature nought but simple existence, motion, and natural exigency; but that metaphysics comprise in addition both intention and idea."

"The science of physics has for its object the research after the *material* and the *efficient*, and that of metaphysics the inquiry concerning *form* and *consequence*.

"The true distinction between these sciences must, therefore, be deduced from the nature of those causes, the discovery of which is their special object.

"What is form? note its interpretation in the *Dictionary of Trevoux*. 'The *form* of an object is that which renders that particular object what it is, and different from all others.' "

Here, then, are the differential characteristics which separate the two orders, physical and metaphysical, even when they are united in one common system. Unassisted reason alone, enables us at once to distinguish these two species of agency:—on the one hand, *materiality* of the cause; infallible and necessary *efficience; progress* in the succession of phenomena; *complete isolation* or indispensable combination: on the other hand, *invisible and inappreciable power*, *design*, *consequence*, *ideal tendency*, and connexion between the successive phenomena, not the result of necessity, but with reference to design, and as a consequence *contingency*.

It is easy to perceive that the distinguishing features of the metaphysical order have been chosen from that special cause belonging to this order, of which we know most, namely, the *human mind*, in which we are conscious of the existence of all that I have just described. But it is equally evident that the same characters are to be found in all living beings that are destitute of the powers of thought, and even of the sentiment of consciousness. The most simple example of life, that, for instance, of the vegetable, suffices to demonstrate in all such objects, an invisible, incomprehensible force, having but a temporary duration, which in its progress accomplishes certain acts primordially imposed upon it, assumes from first to last a configuration conformable to its species, and which, in the course of those vicissitudes to which it is liable from the nature of its surrounding media, acts in accordance, not with the laws of primitive necessity, but with the circumstances of the moment, in order to attain the normal term of its existence.

Here, then, we have the facts on either side,—here do we see the foundation for that division of the sciences in the Philosophy of Nature, of which I lately spoke. Against this division, the materialists, it is true, have revolted; they call it *religious*, *theological*, and if you know aught of their language, you will naturally think such qualifications to be equivalent to an accusation of superstition. But they are in error, the distinction is purely philosophical, and utterly independent of all future intention, whether proximate or remote. It is, moreover, indispensable, since the facts which it comprehends are too diversified to be attributable to one single species of cause. Science at large must be renounced, if a diversity of effects may in every case be referred to the influence of one special agency.

Natural Theology is a human science, which must be placed at the head of all metaphysics, and which the soundest judgments have invariably distinguished from the *spirit of faith,* inasmuch as it proceeds solely from observation and induction, and never from inspiration or enthusiasm.

Next in order to natural theology must be ranked psychology.

I refrain from addressing myself here to those who, refusing to distinguish between the physical and the moral element, support the doctrines of materialism against all, and pretend that thought is but a product, in fact, a secretion. Such belief is unsusceptible of discussion. The association of facts so incoherent in themselves, borders truly upon extravagance. As for chemists, organicians, physicians, who seek after truth, but feel distrust and repugnance for the term metaphysics, they would, I am sure, readily accept the same upon carefully and candidly examining its legitimate signification.

They must be well aware, for instance, that psychology, or the science of the human mind, cannot be placed in juxtaposition with sciences of a Physical Order, but must be assigned such a position as shall render it inaccessible to the latter. Be the interval as wide as you please, the limits are still within the compass of nature.

In this interval, which separates the Science of the Intellect on the one hand, and that of Physics on the other, all is not a desert. Stages are to be found bearing sufficient resemblance to the former to be included within its domain, as subject to its laws, speaking its language, and following its customs.

The characteristic ideas entertained by the ancients relative to the kingdoms of nature, and which Linnæus has so clearly delineated, are well known. Those objects not possessed of life—objects which we no longer dare to style *azotes,* since chemistry has appropriated the word in an acceptation altogether irrevocable—objects, however, which we may be permitted to call *abiotes;* these, I say, *grow; plants grow and live, animals grow, live, and feel.* Buffon continues the progressive movement, and says, *Man grows, lives, feels, and thinks.*

In order to render the formula as concise as possible, the terms *growth* and *increase* have been employed, as applicable to each division; and yet we know that the term *growth* does not express the same process in the different kingdoms of nature. Without, however, dwelling upon this want of accuracy, let us observe the terminal points of the above progression, together with the intermediate stages.

In the *physical order,* then, we have a definite object—infallible action, invariably proportioned to certain evident properties—power always acting *en masse* without reserve—phenomena either entirely isolated and independent of one another, or associated by necessity.

In the *metaphysical order,* wherein is included psychology, we have a cause invisible and inappreciable—variability of action—out of proportion to all manifest causes—a power spontaneously varying in point of activity from absolute latency up to actual violence—phenomena always *associated,* not from inevitable necessity, but in *accordance with design,* and in virtue of a connexion with the past, the present, and the future.

To which of these extremes do the intermediate sciences pertain?

The Cartesians have strenuously endeavoured to include vegetable and animal life, and, as a consequence, the zoonomic life of man, in the physical order; but after exerting themselves for two hundred years in order to justify this attempt, they have not advanced one step: we are forced, therefore, provisionally at least, to regard these causes as experimental agencies requiring direct investigation.

Upon glancing over the characteristics which distinguish the essential cause of all vitality, whether animal or vegetable, we see many that mark in general the *order of metaphysics*. A unity or *personality* which renders two beings incommunicable,—a special tendency implanted in the aggregate, from its origin to its termination, in virtue of which it performs those functions which had been assigned to it at the creation of the motor power,—a rule of conduct (if we may so speak) rigidly adhered to, in spite of obstacles which render its observance difficult if not impossible,—a *vis medicatrix*—a spontaneity which allows the said power to vary its mode of action, and to yield to external impressions only as far as may be desirable.

Would you then construct new Sciences with such materials? I ask to what known Science would they bear any relation? In Psychology alone will you discover similar laws; there only will you find a dictionary, the terms of which really express the truth; in it alone will you find appropriate formulæ. Let us, therefore, assign these sciences a place in the domain of Metaphysics, and in close proximity to Psychology, where they cannot fail to discover analogy and consanguinity.

It is needless to say that human biology deserves precedence, whether by right of parentage or of association.

Before the time of Descartes, philosophers taught that bodies to which the term *abiotes* is applied, acted *ratione entis*, and that living aggregates acted *ratione moris*. This language was also adopted by Arnaud, while the followers of Hippocrates have likewise preserved this distinction. It is evidently identical with the distinction between the sciences of the *physical* and those of the *metaphysical order*.

You are perhaps aware that the spiritualists of the non-medical community have restored the almost forgotten worship of Descartes, because this philosopher was one of those who advocated with the greatest amount of zeal and plausibility the distinction between material and spiritual substance on the one hand, and the spirituality of the mind on the other.

None can more thoroughly appreciate than myself the services which Descartes rendered to philosophy, mathematics, and ethics. But being unwilling to recognise more than two primary causes, Matter and Spiritual Intellectuality, he has of necessity classed in the *order of physics* all causes not essentially intellectual. Thus, the vitalism of Hippocrates and his followers, has been utterly proscribed in this philosophical revolution, and it is not the fault of Descartes that legitimate medicine itself remains unscathed. Happily his endeavours to explain life upon mechanical principles have been so transparent and futile, that the doctrine and its supporters are all but extinct. Our school, therefore, has but little cause for self-laudation as respects this scientific event. If it has encouraged anatomical studies and opened up the more mechanical portions of functional physiology, it has thrown discredit upon the true principles of Biology in general, and of Human Biology in particular.

It is by no means improbable that this resuscitated enthusiasm for Descartes may have appeared to the Organicians a favourable opportunity for replacing biology among sciences of the physical order. They dare not reject the term *physiology*, but endeavour silently to bring about a Cartesian interpretation of the same; and as we are now seriously engaged with the duality of the Human Dynamism, and have been striving to establish an accurate distinction between the zoonomic life of man, his intellectual existence, and the laws of physics generally, I deem it right to guard you against the stratagems employed by the materialists, to erase the characteristic features of Human Biology.

An example of each may be found in the tendency which certain authors possess, to use, indiscriminately, terms pertaining to the *physical order*, when treating of a science belonging to the domain of *metaphysics*.

The sect of the Organicists, who seek to discover the whole human dynamism in the anatomical study of the system, would wish us to follow their example. True it is, that medical legislation has very properly imposed the manual duties of the dissecting room upon our students; but our rivals, not content with this, would take advantage of the salutary regulation, to confine us within its sphere, and to render the transgression of its limits impossible.

They pride themselves upon the title of *Anatomical Physicians*, and exert themselves to the utmost to prevent their opponents from being aught else.

Their conduct on this head, appears to me to bear a strong resemblance to a picturesque and somewhat epigrammatic composition emanating from the pencil of the celebrated painter De Retsch. A traveller speaking of his productions says, "Some of his sketches possess all the piquancy of caricature without its satire. In one, for instance, the Genius of Arts, under the aspect of a young Apollo, is condemned by Ignorance, Vulgarity, and Folly, to grind a hand organ. His empty purse lies at his feet, his palette and lyre are broken, and he raises towards heaven a countenance indicative of abstraction and sorrow; this allegory is really a *chef-d'œuvre!*"

It would be an easy task to parody the above. Let us suppose an individual desirous of studying the science of man in its fullest sense, but condemned by Organicism to abandon the more elevated and abstruse departments of the subject, in order to spend his time upon mere manual exercises. Let the persecuted man still preserve, if you will, the features of Apollo, this divinity is as much the emblem of medicine in its noble, scientific, and elevated form, as of the fine arts. His allegorical enemies might be, not indeed *ignorance*, but *narrow-minded anatomism;* in place of folly, *evil intention.* These divinities oblige their victim to spend his time in dissecting a corpse, or in performing experiments upon a frog. It would not be difficult either to imagine a sign of indispensable necessity, as typified by the empty purse. The works of Hippocrates and Bacon are thrown aside, torn, and neglected.

Scenes of this kind are not very tragic; and I would merely draw a moral from the above. It is this, that the Apollo, be he an artist or a physician, should yield courageously to necessity, whatever it may chance to be; that he break or cast aside none of the tools he may have been forced to employ, since there is nothing which may not be turned to account sooner or later; and that, lastly, when the necessity is past, the best course he can adopt will be to mend, as far as possible, his lyre, his palette, or his books, and to renew his studies, in spite of the enemies who may have oppressed and constrained him.

This leads me now to say a few words, respecting the social position of our art and its estimation in public opinion.

So great is the importance of medicine as a science, that its due appreciation ought, in my opinion, to form a portion of public morals. But you must understand that I employ this term in the sense of Duclos,* as the *ensemble* of a nation's opinions, upon a subject which may influence its mode of thinking, feeling, and acting, not only in regard to the subject itself, but likewise as respects the individuals who may be its depositaries.

If the public, however, are to entertain just ideas relative to the science of medicine and its professors, it must be through the medium of an education, in which would be included the essential dogmas of science, and the more rational and general deductions of art.

Public instruction, however, has never been based upon such a method. But would it be difficult to comprise within the limits of what is usually designated a liberal education, the information necessary to attain such an object? I think not. I even venture to assert, that if those to whom is deputed the task of teaching philosophy, zoology, and botany, would but, in the discharge of their duties, disseminate among their hearers a few anthropological truths, . . . . the rising generation, cognizant of these truths, hitherto restricted to our schools of medicine, might become capable of hazarding an

* Considérations sur les Mœurs, chap. 1.

opinion upon the agitations which occasionally disturb the medical community.

This community is, as we well know, very prone to internal dissensions; of these the lettered public know but the resulting enmities and scandal. Why, however, should it not be able to appreciate their radical source, which is rarely, indeed, an important discovery, but the ignorance of some fundamental principle, or a defect of logic easily verified.

It is not my intention here, to present you with a list of those medical truths which might advantageously form a portion of liberal education, without danger of cramping the mind by long pedantic specialities; but I do not hesitate to mention a few of those ideas which have been already put forth, and to propose them as examples of what might be a useful adjunct to the ordinary course of public instruction.

The constitution of man is, without doubt, one of the most important branches of medicine; but we have seen that the chief facts in connexion with it are:—1st. The distinction between the two powers of our dynamism, as proved by the insenescence of the intellectual principle; 2ndly. The fusi form configuration of zoonomic life; 3rdly. Its collocation in the *order of metaphysics* contrary to the doctrine of the Cartesians; 4thly. The indispensable necessity for a direct and special knowledge of the vital force, as occurring in man.

It is to the teachers of botany and zoology that I look, in order that their pupils may entertain just notions relative to animal and vegetable life, the characteristics of which exclude those of bodies comprised in the purely *physical order*. The works of Cudworth and Grew, analyzed and abridged by Leclerc, are indestructible monuments of logic, subversive of all the efforts of mechanicism.

But it is in the schools of philosophy that the fundamental doctrines of the human constitution may be cultivated and disseminated with advantage to the studious portion of the rising generation: "Know thyself," *Nosce te ipsum*, is a precept applicable to all; but it is not followed when psychology alone is made the subject of investigation; an aggregate material deserves equally to be studied. When treating of the connexion between the intellectual principle and the organic system, I am convinced that the hypotheses of physical pre-determination, and of pre-established harmony, are mentioned merely as historical portions of the science. But it affords, nevertheless, a good opportunity for inculcating the reality of an intermediate vital principle, which is neither of intellectual nor physical origin, and thus to demonstrate the duality of our dynamism. Of this, the insenescence of the intellectual principle, in spite of advancing years, is one of the simplest and most convincing proofs.

The progression and general configuration of the duration of man's vital principle, readily admits of a comparison between this principle and that of the beast. I see no need of very profound researches to demonstrate the difference which exists between the human dynamism, which is dual, and the animal dynamism, in which no such duality is apparent, for want of the characteristic of insenescence.

Equally susceptible of demonstration in the animal, is the all-powerful nature of the vital force, which entirely controls it; and in man, the precarious condition of this same principle, which, apart from those economic and automatic functions which it exercises by its own unaided powers, appears feeble, subjective, and submissive, capable of much, but only in compliance with and under the direction of a higher authority.

The possession of just notions relative to the constitution of man, would, I conceive, teach the public the different aspects under which members of the industrial, artistic, and professional community, severally minister to our inevitable exigencies. The public know full well that the Moralist, the Professor, the Theologian, &c., have for their mission the correction and perfection of my intellectual powers, since it has long been taught that soul and body are not identical; but it knows not to which of my elements the Physician, the Artist, the Empiric addresses himself; these it classes together under one category, and for this simple reason, that it ignores the fact of there being in the living system of man a principle of metaphysical origin and order, which unconsciously and silently presides over and regulates each individual organ. Were professors of philosophy but willing, the future public would not fall into errors of this kind. After a few colloquies, it would discern the vocation and aptitude of the artist without consulting his diploma, and would soon discover the man whose studies had been limited to the contemplation of one or more organs, . . . . . . the man whose attention had been directed to the manifestations and tendencies of that creative power which regulates and preserves the integrity of those organs, and he who, a stranger to all this, would act and counsel by mere chance.

You may, however, probably imagine that it would be no easy matter to persuade the frequenters of our schools of philosophy, that the duality of the human dynamism is an actual fact; you may think, moreover, that such a dogma requires long and laborious induction, and is to be developed only by the members of our profession. But you mistake; for while this principle is directly deducible from the fact of our intellectual insenescence, I may add that the educated portion of the community have on this point an inkling, so to speak, of the truth. I propose to devote a portion of my lecture to the proof of this assertion.

Already have I shown you how the public has tacitly accepted the psychological Agerasia before it had even been clearly demonstrated. I conceive that it does exactly the same in reference to the duality of the human dynamism.

1st. We find in certain of our dictionaries a distinction drawn between the *spirit of vitality* and the *spirit of intelligence.* In that of Trevoux, we read under the word *life,* the following: "The expression, life, is also employed in " speaking of the constitution, of the principle of heat and motion which animates " bodies, and induces action, feeling, growth. It is sensitive and animal exist- " ence . . . . . . God . . . . breathed the breath of life into the body of Adam."

Of the meaning of the term *spirit* in Psychology, you are already aware. It is " the sum total of the mental powers, or rather the mind itself, as endowed with conception, judgment, imagination, and self-consciousness."

Public opinion, then, we see admits two series of phenomena, and two series of causes. The phenomena ranged under the causal denomination of *Spirit of Life,* are such as we designate *vital, natural, automatic.* Those classed under the title of *Soul or Spirit of the Intelligence,* are what we on the other hand term the *intellectual faculties.* By distinguishing causes from effects, the public has decided that those causative agents which differ from one another, should be likewise distinguished by names indicating that neither pertains to the order of physics. *Spirit of life, spirit of intelligence,* do not these words express causes as distinct as the effects to which they give rise? But the term *Spirit* belonging alike to the two denominations, teaches us that these two causes belong to a class apart and distinct from that which comprises all objects destitute of vitality.

2ndly. You may perhaps have remarked that the above distinction is indicated in the most ancient book on record,—the Book of Genesis.

We here perceive, that in the formation of man two distinct acts are recorded. Thus, in the 2nd chapter and 7th verse, we read—" And the LORD GOD formed man of the dust of the ground, and breathed into his nostrils the breath of life, and man became a living soul." Up to this point the material aggregate possesses but a zoonomic existence; we require another divine act before the dawning of intellectual life. The learned Grotius has similarly regarded the details of this narrative. To complete it, a separate verse explains the creation of a *spiritual existence after the likeness or image of the Creator;* and its dominion over all other created beings.

It is impossible in this narrative not to recognise the idea of the duality of the human dynamism. Whatever its origin, it is intimately connected with every view we may take of the constitution of man.

3rdly. In regarding this text attentively, you will be surprised at finding a contradictory passage in a work regarded by connoisseurs as a *chef-d'œuvre;* I allude to Colbert's Catechism of Montpellier. After the question "How did God make man?" we find the following answer: "*He formed his body from the earth and gave it life by uniting it to a reasoning spirit. For it is this which really constitutes the essence of vitality in a human body.*" None could speak thus that were not imbued with the doctrines of Descartes; none but a follower of his could thus designate life as being nought but the mode of action of an intelligent principle, and at the same time regard the existence of the animal as the mere working of a machine. You will notice that Grotius, who recognised the duality of the human dynamism, died before Cartesianism had attained its maximum of influence, and that Bishop Colbert, as a staunch Jansenist, considered it necessary to adopt the opinions of Descartes, for whom the Jesuits had but an equivocal and vacillating affection. But let me ask, is it possible to assert that an infant born without brain and spinal cord, lives in virtue of its intellectual capabilities?

Think not, however, that I wish to oppose that Christian ordinance, which decrees that the rights of baptism shall be granted even to such a being as I have just mentioned. We may safely obey a law which has been instituted by superior authority, as at least erring on the right side, and without attempting to decide upon facts beyond its province.

Here I may perchance be permitted to offer an idea which suggests itself to my mind, of the value of which my hearers alone can be the best judges.

You must be aware then of the celebrity of Haydn's Oratorio, entitled "The Creation." The author of the libretto was an illustrious physician, and at the same time a tolerable poet and good musician, viz., Von Sweiten, physician to the Empress Maria Theresa. In it there is a scene, where the angel Uriel describes the creation of the man and the woman.

> Dieu fit à son image
> Son plus bel ouvrage
> Sa volonté d'un souffle anima;
> L'homme et la femme qu'il forma
> Il voulut leur donner une ame,
> D'un rayon de sa flamme
> Une etincelle l'alluma.

Spite of the imperfections of this history, I recognise the very points which I desire to impress upon your minds. First you perceive the general fact, and then its subdivision into distinct portions; to wit, the *vivification* of the man and the woman, and subsequently the addition of a reasoning mind to the collective system. I shall not here stop to examine whether it be lawful for the poet to have created and vivified the sexes, previous to the endowment of reason; what chiefly interests me is this, that I perceive the elements of the dynamism to have originated separately, and in proper order. Von Sweiten has faithfully interpreted the physiological meaning of the Mosaic text, and this is the more to be admired in a votary of Boerhaave, who had once been a Cartesian. True, the master had, in his old age, become a disciple of Stahl, nor am I surprised that the pupil should have, in process of time, re-entered the Hippocratic pale.

4thly. The notion respecting the duality of the human dynamism, appears to have been vaguely expressed in the pictorial representations which the early Christians were in the habit of carving on the sarcophagi of the catacombs.

In looking over the plates in Bottari's *Roma Sotternanea*, I have remarked several drawings of tombs adorned with bas-reliefs, the subjects of which bear reference to the Christian's hope. One of the most common of these symbols is the history of a miraculous resurrection—that of Lazarus, for example. It is represented in different places by a species of mummy encased in swaddling clothes, and placed in a niche; but I do not remember to have recognised in the engravings, either the daughter of Jairus, or the son of the widow of Nain. Wherefore then this preference for the miracle in question, over similar ones recorded in the sacred writings? Simply because the most convincing of these supernatural transactions is that in which death is most evident, and in this respect, the case of Lazarus was all that could be desired. For four days he had ceased to breathe; he was buried, and at the opening of his tomb, the evidence of decomposition was strikingly manifest, *Jam fœtet.*

The choice of this resurrection as a subject of artistic representation is sufficiently rational to afford grounds for believing it to have been intentional. The artists had, I conceive, a notion of *apparent* death, and imagined that in this condition a spirit of vitality might preserve the body from corruption during the absence of the intellectual principle. The incredulous have asserted that the resurrection of Jairus' daughter and of the widow's son were merely recoveries from asphyxia, a re-union in fact of the vital force and the intellectual principle, and consequently by no means supernatural. The sculptors, however, have contented themselves with representing an occurrence, the miraculous nature of which was beyond all controversy, alike convincing to the Pharisees who denied the power of Christ, and to the Sadducees who denied the soul's existence after death. The death of Lazarus was complete; the odour of the body proved the extinction of that quiescence which is the *ultimum moriens* of the vital faculties. Allowing that the artists in question were incapable of devising a pictorial formula which might represent the distinction between the two principles, as we have seen may be done in the Hippocratic School, it cannot be doubted but that they entertained a confused notion of this duality, without which, they never would have recognised the two kinds of death,—1st, incomplete death, in which the erratic spirit may yet be recalled; and 2ndly, complete death, in which the spirit is irrevocable, and where the extinction of the vital force shuts the door of hope, since that extinction is accompanied by a rapid destruction of its previous tenement—the body.

I think, moreover, that I see in the Christian faith, an idea resulting directly from this sudden decomposition of an actual corpse. According to Scripture we have no grounds for questioning the complete death of Christ, since upon this condition only can his subsequent resurrection be considered miraculous. But all was pre-ordained. If the body of the Saviour when buried, did not undergo the fate of that of Lazarus, it was because, according to the Church's interpretation, a divine power fulfilled the part of the extinct vital force; a miracle which had already been predicted by the Psalmist. "My flesh, therefore, shall rest in hope, for thou wilt not leave my soul in hell, neither wilt thou suffer thine Holy One to see corruption!" Thus, in prophetic language, *intellectual existence* will cease; there will be complete death, and there must therefore be some divine substitution at hand, to replace that natural cause of *metaphysical origin* which had now become exhausted and extinct through physical suffering and hæmorrhage.

5thly. The records of Pagan antiquity are replete with ideas suggestive of the duality of our dynamism. The fable of the man formed by Prometheus is accompanied by circumstances which plainly indicate the prominence of this dogma in the minds of the artists of those days. Prometheus had fashioned the material aggregate with all the skill of which a secondary deity might be supposed capable. But the system was still but an inanimate body. Minerva, wiser than Prometheus, and wishing to reward him for certain services, gave him advice; drawing near then to the precincts of the immortal gods, the artist stole a spark of fire with which he vivified his statue. Hence arose that which at a later period was designated as the *calidum innatum*, or vital force, which we regard as the principle of zoonomic existence. But what was this fire? was it one of the sun's rays? No, it was of the physical order, and the greatest of the Poets imagined that this vivification could only have taken place through the intervention of something, itself virtually endowed with vitality. To complete the myth, Hesiod asserts that Prometheus actually stole the fire from Jupiter himself, who had previously hidden it, and then made use of it in order to vivify his own handiwork.

But what was the result of all this? was it a man? No! the statue had been endowed with life, but not with reason. To crown the work, Minerva comes and places on the head of the newly created being, a butterfly, the emblem of intelligence. Then, indeed, the man is complete. To frame the above fiction, it is evident that its authors must have possessed intuitively that knowledge of the constitution of man, which we derive from demonstration.

An able writer, the Abbé Guerin du Rocher, has published a work entitled "*A True History of the Fabulous Epochs.*" Many of the fables it contains would be inexplicable without our present knowledge of the duality of the human dynamism. The theology of paganism, in reference to the *manes* and *lemures*, to the doctrines of *metempsychosis*, and to certain forms of *apotheosis*, is evidently founded on this anthropological fact. The application of the duality of the human dynamism, to the interpretation of these religious observances, may serve to prove that since the origin of philosophy up to the present time, the mind of man has been imbued, more or less, with the idea of an existing distinction between the two causes of *metaphysical origin* which animate man.

Whatever, then, may be our private views relative to the fabulous narratives of antiquity, it would be unwarrantable to regard as fanciful and unmeaning, ideas which have formed the groundwork of venerated usages, of religious institutions, and of moral law, among nations enjoying a high degree of civilization. I do not, therefore, hesitate to insist still further upon certain reasons for recognising the adoption, whether implicit or explicit, of the dogma of the duplicity of our dynamism, among the ancients.

You are doubtless aware of the worship of the *Manes*, and that the term *Manes* was intended to signify the immaterial part of every human being, in contradistinction to the *exuviæ* or mortal remains which were to be consigned to earth. I refrain here from entering upon the history of all the dogmas connected with this species of worship among the Romans, and will confine myself to a single idea upon which I would wish my auditors to fix their attention. The term *Manes* is not a collective one, intended to represent the souls of the dead in general. The immaterial portion of a single individual bears the same name in the plural.—But why in the plural, when one man only is spoken of? I have frequently propounded this question, but have never received a satisfactory answer.

If we bear in mind the opinions of the ancients, as expressed in their myth of the man formed by Prometheus, we may fairly presume that the dynamism, when separated from the material aggregate at the moment of dissolution, was, in their eyes, dual, and that the two principles of which it was composed had separate destinies.

Delandine, in his work, entitled, "*L'Enfer des Anciens*," says that "The altars which were erected to the *Manes* in Etruria, Calabria, &c., were always two in number, and placed very near to one another;" he does not, however, assign any cause for this custom. But it tallies so completely with the idea of duality, that I cannot but think the plurality of the term and of the altars themselves, to have been the offspring of the same belief.

What then became of the two metaphysical causes after death? According to the doctrine of the ancients, the spirit was for ever doomed to Hades, unless, after a sufficient period, it drank the waters of Lethe and returned to the upper world. Except in this case, the soul could not transgress the limits of Pluto's empire. The exceptions made in favour of Eurydice and Alceste, only serve to confirm the rule. True it is that *evocations* were sometimes attempted; but the hope of a supernatural manifestation never engendered a belief that the soul thus solicited could effect an emigration or change of locality.

Divine offerings, nevertheless, were habitually made to the manes of beloved departed friends and relatives; these were presented for the most part in the evening, about the close of twilight. We may here ask, did the object of either altar profit alike by these offerings? Or did one of the invisible principles possess the power of coming and hovering near the common tomb, while the other remained in Tartarus to suffer condemnation, or enjoy reward?

We require some information on this head, to clear up the chaos which constitutes this portion of Pagan Theology.

Again, what were the *Larvæ* and the *Lemures* that were held in such respectful awe by the Romans, and for which they instituted nocturnal fêtes, celebrated in the month of May? They were malevolent beings who troubled the repose of the living, not unfrequently seizing them, and depriving them of reason (Plautus). It was the *Lemures* and *Larvæ* that animated the skeletons of those pictorial representations, which we find upon ancient monuments (Lessing). The poets of the middle ages have reproduced this fiction, in order to compose the dance of the dead.

Now may not the evil spirits designated in ancient history, by the terms *Larvæ* and *Lemures*, be nothing more than the pernicious instincts which are inseparable from the human vital principle, and irresponsible? since the power of which they are but manifestations is not endowed with a sentiment of consciousness . . . instincts, I repeat, which constitute what the learned have designated by the word *morositates*, and which the public have regarded as *eccentricities*. The body medical has long since acquainted the legislature with the fact, that there exist individuals in whom the component parts of the dynamism are so at variance the one with the other, that the intelligence struggles perpetually against the detestable suggestions of a morbid vital principle. The reasoning powers repress and abhor, in one a homicidal tendency void of purpose, in another a disposition to purloin, in another a tendency to general destructiveness, in another a tendency to lavish affection and solicitude upon objects totally unworthy of such sentiments. It is these states of mind which border so closely upon actual folly, but which must nevertheless be distinguished from mental alienation, since the principle of intelligence possesses its full measure of integrity and rectitude, and condemns with as much sense of humiliation as of justice, not merely the propensities against which it strives, but even those to which it succumbs.

There are certain conditions of the mind, characterised by a tendency to imitate the habits of animals, whether savage or domestic, and termed zoanthropy, lycarthropy, cynanthropy, hippanthropy, which date from the earliest period of fabulous history, and have been known since the very foundation of medicine. It would appear that the Greek word *morosophia* was expressly used to designate such conditions of the intellect, in which an individual presented the singular coincidence of sanity and aberration; in other words, the anomalous condition of reason not possessing sufficient power to repress extravagant tendencies.

Those persons who in ancient times fulfilled at once the functions of priest and physician, might have instructed the legislature in the duality of the human dynamism, and the hierarchical order of the two principles. The priest and the legislator might have said:—"Since the dynamism of man is dual, since the manes are recognised as being so likewise, and since on quitting its human habitation, each component part finds an altar ready prepared; it becomes necessary that a tribunal should pronounce on the merits or demerits of that principle which is endowed with reason, and that its recompence should at once be granted, or its punishment inflicted. As regards the remaining principle, which is unsusceptible of blame, and merely obeys, blindly, those inward impulses which are either of simultaneous origin with itself, or are acquired under the influence of external circumstances, it may wander without restraint, either in hades, or on the earth itself, since its destiny has been fulfilled. The Larvæ, the Lemures, &c., may still be of some utility on earth, inasmuch as they will serve to maintain a species of religious sentiment, not without its value in society."

It is by no means improbable that the sentiments of apprehension relative to these spectral beings, may have been carried to a great extent by those who had never reflected upon the decay of the vital principle subsequent to its culminating period. Were we ignorant of its progressive declension, we should have as much ground for believing in its immortality, as we have for the like evidence in respect to the soul.

10. Albeit the opinion respecting the doctrines of metempsychosis may not have constituted a separate religion existing under the sanction of political authority, it has nevertheless been taught by moralists of high standing, and has been long maintained by certain sects. It is indeed another event of which the student of philosophy should not merely take cognizance, but should also inquire into its most elementary notions.

You may easily understand how that in a religious belief of this description, I can merely discuss those points which bear upon the constitution of man. I omit, therefore, all collateral ideas relative to this topic, that I may not have to reproach myself with causing you to lose sight of the essential objects of our obligatory studies.

Let me, therefore, beg of you to fix your attention for a moment upon the belief in question.

The manner, then, in which Herodotus describes the Egyptian metempsychosis, undoubtedly pre-supposes that the living being is possessed of two simultaneous principles; the one vital, the other psychical. "On quitting the body," he says, "the soul enters another animal body, already prepared for it, and when it has passed through every species, whether terrestrial, aerial, or maritime, it again enters a human body; and this cycle is accomplished, according to the Ægyptians, in 3000 years." It is, I think, clear therefore, from this, that the soul could not have been regarded as the means of imparting vitality to the animal, which was already born, and consequently living. The psychical principle was but an adventitious element. It had become connected with the material principle by *hypostatic* conjunction.

11. Another example, which proves that our knowledge of the constitution of man has been for a length of time consigned to the various departments of literature, more especially that which relates to archæology, may be drawn from an ancient basso-relievo, the subject of which is an apotheosis, according to the ideas of the Egyptians.

This basso-relievo represents the ascension to heaven of a soul, just emancipated from its earthly tenement. On my right I perceive the apparel of a tribunal that has passed sentence on the conduct of the individual during his life-time. The decree has been favourable, and the corpse, legally embalmed, has been consigned with due honour to an elegant but chaste sepulchre. The coffin is placed upon an inclined plane, and the learned commentator says, that in such funeral solemnities, the body remained in this position for one year. The soul that has just left it, is amid the celestial regions; it is represented, I should add, as still retaining a human configuration. The lower half of the figure is visible in space; the upper half is in the bosom of the Deity. The line of separation is about the region of the kidneys, the thighs and inferior extremities being alone discernible. Numerous stars surround the figure with a glorious halo, expressive, doubtless, of the good and brilliant qualities which had adorned the individual when on earth. Within the tomb itself, may be seen a star, having somewhat the appearance of a comet, and differing in its character from those which are presumed to represent the moral qualities of the deceased. It is, in fact, no longer a mere light, but a cause of a more consistent, tangible nature, remaining in close proximity to the body which it has nevertheless quitted. The commentator regards it as the *good genius* of the individual; now, inasmuch as provided we agree upon things, I care little for their names, the one in question pleases me as well as that of ***instinct, vital force, spirit of life, essence of vitality, &c.*** Each and every one of these terms expresses alike the idea of a metaphysical agent, which is not the intelligence on the one hand, since that has flown upwards into the regions of space, nor the material system on the other, inasmuch as this still remains enveloped in its funereal trappings. The archæologist had not the same ideas respecting the nature of man as we have, and saw only moral excellence in this emblem; we, however, whose physiological knowledge is traditionally united with that of the ancients, are better able to interpret the meaning of the symbol, which is too far removed from the soul to be of a psychical nature, and sufficiently near the corpse to have but just quitted it.

These remarks may suffice to show that our notions respecting the constitution of man, and more especially those which relate to the duality of his dynamism, are not entirely foreign to the ordinary credence of the public at large, in all ages . . . . . . inasmuch as they would appear to be intimately connected with the study of the Belles Lettres, and of History, Philosophy, and Literature generally, whether ancient or modern, domestic or foreign.

Nought to my mind would be easier than to inculcate among the rising generation those exact unadulterated medical truths, which constitute the very basis of the science of man; and at the same time, to place in juxtaposition with such truths, those allegorical, hypothetical, and poetical adumbrations, by which they have at all times, and in all places, been more or less enveloped. This would, I think, suffice to place us in communication with all the votaries of literature, and would enable us to appeal to their judgment in cases where the prejudice or partiality of our medical brethren might induce them to censure both ourselves and our doctrines without sufficient reason.

Think not, however, that I hope, or even wish, that an enlightened public should comprehend the more abstract propositions of medical physiology. No, we should no more expect this, than we should anticipate a popularisation of the differential and integral calculus, of the mechanism of the starry heavens, of the details of geology, or of many other sciences, for which a special vocation and profound study are essential. But I may be permitted to hope that the elementary principles and essence of our doctrines shall one day be considered as necessary as the rudiments of mathematics, chemistry, natural history, and other sciences which are certainly not of more importance than that of anthropology, and sincerely do I trust that this wish may be realized.

